# Felt presence and its determinants in young adults: Results from three independent samples

**DOI:** 10.1192/j.eurpsy.2025.1992

**Published:** 2025-08-26

**Authors:** J. Maciaszek, M. Rejek, T. Bielawski, M. Błoch, A. Senczyszyn, B. Misiak

**Affiliations:** 1 Department of Psychiatry, Wroclaw Medical University, Wroclaw, Poland

## Abstract

**Introduction:**

Felt presence (FP) is a phenomenon that might appear in individuals with mental and neurological disorders as well as those without any specific morbidity. Some studies have indicated that FP is closely related to psychotic symptomatology. Yet, the mechanisms underlying its occurrence remain largely unknown.

**Objectives:**

The present study aimed to investigate whether FP is associated with widely known risk factors of psychosis.

**Methods:**

Data from three independent samples of non-clinical young adults were analyzed. Self-reports were administered to assess psychopathological symptoms (samples 1 – 3), neurodevelopmental risk factors for psychosis (sample 1), social defeat components (sample 2), childhood trauma, and loneliness (sample 3). A total of 4782 individuals were surveyed across all three samples.

**Results:**

Unadjusted analyses showed that the following factors are associated with higher odds of FP: obstetric complications, childhood trauma, non-right-handedness, a lower education level, unemployment, minority status, humiliation, perceived constraints, and loneliness. However, only minority status and a lower level of education were associated with higher odds of FP after adjustment for other psychopathological symptoms, age, and gender. Importantly, hallucination-like experiences across all recorded modalities and paranoia were associated with higher odds of FP in all samples. Depressive symptoms were weakly associated with FP in two samples.

**Conclusions:**

Findings from the present study suggest that the majority of known risk factors for psychosis contribute to the emergence of FP through the effects on psychotic experiences. Low educational attainment and minority status might be the only risk factors independently contributing to the emergence of FP.

**Disclosure of Interest:**

None Declared

